# Influence of lip position on esthetics perception with respect to profile divergence using silhouette images

**DOI:** 10.1186/s12903-023-03537-3

**Published:** 2023-10-24

**Authors:** Abdulrahman K. Alshammari, Muteb A. Algharbi, Sulaiman K. Alshammari, Ali A. Alenzi, Yasser R. Malik, Malik Z. Abideen, Ammar A. Siddiqui, Ahmed A. Madfa

**Affiliations:** 1 Department of Preventive dentistry, College of Dentistry, University of Ha’il, P.O. Box 2440, Ha’il, Saudi Arabia; 2 Emergency Care Center-RU, Ministry of National Guard Health Affairs, King Abdulaziz Medical City, Riyadh, Saudi Arabia; 3 Department of oral and maxillofacial surgery, Prince Mohammed Bin Abdulaziz Hospital, Riyadh, Saudi Arabia; 4 Department of Dental Education, College of Dentistry, Bakhtawar Amin Medical and Dental College, Multan, Pakistan; 5 Department of Restorative Dental Science, College of Dentistry, University of Ha’il, Ha’il, Saudi Arabia

**Keywords:** Aesthetic perception, Facial profile, Lip attractiveness, Maxillofacial Surgery, Orthodontics, Saudi Arabia

## Abstract

**Background:**

The aim of the study was to determine the facial divergence and lip position combinations that are most and least preferred, and to investigate whether age or gender has an impact on these preferences.

**Methods:**

The current investigation was carried out on a sample of 1077 individuals who were not experts in the field (253 men and 824 females). The research employed black silhouette photographs of profiles featuring different lip locations and profile divergences. The recruitment of participants was conducted in order to assess the attractiveness of the profiles, employing a Likert scale. The various positions of the lips and variations in facial profiles were thoroughly categorized. Results were analyzed using the Chi-square test.

**Results:**

The findings of the research demonstrated that aesthetic perceptions displayed diversity when considering different lip locations and profile divergences. It was shown that neutral lip positions were predominantly favored, accounting for approximately 40.2% of the total frequencies in the anterior diverging group. It is noteworthy to highlight the aesthetically pleasing features exhibited by those with the most prominent lip position, occurring at a frequency of 10.9% in straight-diverging group. In the posterior divergent group, the most protruded lip position, showed very attractive aesthetics with frequency (7.1%). Gender, age, region, and level of education had significant influence on aesthetic perception.

**Conclusions:**

The variety of aesthetic preferences is influenced by the location of the lips and the divergence of the facial profile, resulting in different outcomes within the categories of anterior, straight, and posterior divergence. Clinicians are advised to customize the treatment regimen in order to correspond with the unique desires and preferences of the patient.

## Background

In recent years, there has been a significant focus on the aesthetic aspects of facial harmony and attractiveness. The current society has shown significant interest in the aesthetic perception of the face, irrespective of its structural or functional aspects. It has been shown that individuals seeking orthodontic treatment are predominantly driven by their own self-perception of dentofacial esthetics [1–4]. One of the primary factors motivating individuals to get orthodontic evaluation is their subjective perception of their dentofacial aesthetics, which also exerts a substantial influence on their treatment expectations. Nevertheless, the underlying foundation for this self-perception is contingent upon individuals’ self-assessment upon gazing into a reflective surface, wherein frontal perspectives of the countenance and the presence of a smile frequently serve as indicators of their primary preoccupations [3, 5, 6]. The aesthetic aspects of orthodontic therapy, including the soft tissue and its many components such as lip position and facial convexity, play a crucial role in diagnostic and treatment planning [7].

The attention garnered by facial aesthetics extends beyond mere harmony and attractiveness, as facial symmetry emerges as a significant contributing component. The assessment of soft tissue profile esthetics can vary between clinicians and laypeople due to differences in emotions, knowledge, and behavior. These variations contribute to the development of individualistic perspectives. Therefore, it is crucial to evaluate esthetic characteristics from both the layperson’s viewpoint and the perspectives of clinicians, such as orthodontists and maxillofacial surgeons [3, 8–10]. The orthodontic standards that are currently employed, such as horizontal lip position, are frequently more precise in reflecting the anatomical norms of the individual populations for whom they were initially developed [11, 12]. Rather than considering the overall population, earlier studies have primarily focused on averages derived from individuals with acceptable to excellent face traits [13]. It is worth mentioning that the Ricketts E-line seems to rely solely on clinical experience, lacking any accompanying documented sample [12, 14]. Furthermore, Steiner’s sample was carefully chosen by orthodontists who considered factors like as favorable face esthetics and occlusion. The predominant approach employed by orthodontists is the utilization of hard tissue analysis as the basis for their standards, rather than relying on socially constructed aesthetic norms.

Despite the considerable importance of aesthetics, there exists a paucity of research investigations pertaining to this domain. Furthermore, the existing body of research is predominantly characterized by controversial findings and is primarily derived from studies with limited sample sizes [15–17]. One characteristic of facial symmetry that can be altered by an orthodontist is the protrusion of the upper and lower lips [18]. Selecting an appropriate treatment approach that aligns with the patient’s preferences can pose challenges when there exists a disparity in the patient’s and physician’s perceptions of aesthetic profiles [19]. As a result, orthodontic professionals are compelled to incorporate the examination of facial attractiveness into their treatment approach in response to patients’ desire for enhanced facial aesthetics. The potential for patient dissatisfaction exists despite the positive outcomes observed in surgical and orthodontic interventions, primarily due to a lack of understanding regarding the patients’ treatment expectations [20]. One characteristic of facial symmetry that can be modified by an orthodontist is the projection of the upper and lower lips. The positioning of an individual’s top and lower incisors has an influence on their lip posture. Research has indicated that the retraction of patients’ lips is a potential outcome subsequent to the extraction of their incisors and premolars [21].

In addition to providing optimal patient care, it is imperative for orthodontists to objectively evaluate the patient’s expectations through the application of guidelines, norms, and ideal ratios. This is crucial due to the fact that individuals without professional expertise may possess varying notions of aesthetic profiles, largely influenced by prevailing “beauty culture” within their social circles [22]. Hence, it is important to note that achieving positive outcomes in surgical or orthodontic interventions does not guarantee patient satisfaction unless their specific expectations are fulfilled [23].

Lip surgery can be used as an alternative method to reconstruct the function and aesthetics. The surgical procedure mainly used if there are large defect such as a cancer or gummy smile. An optimal method of reconstruction would entail a singular procedural stage such as Karapandzic Flap wherein the defect is substituted with comparable tissue, so reinstating both aesthetic and functional aspects, while also ensuring reliability [24–26].

The primary focus of study in this particular topic is centered around examining the responses of soft tissues to movements generated by orthodontic treatment [27]. Several investigations have established a correlation between soft tissue qualities and horizontal malocclusions. However, only a limited number of studies have focused on the soft tissue components of malocclusions from a vertical perspective. Moreover, the existing literature on this subject [28, 29] frequently fails to conduct a comprehensive analysis of the underlying factors contributing to these disparities or their potential origins. Consequently, there is a necessity to generate additional data pertaining to these morphological categories and their associated soft tissue characteristics. Therefore, the objective of this research was to determine the facial divergence and lip position combinations that are most and least preferred, and to investigate whether age or gender has an impact on these preferences.

## Methods

The Medial Ethical Committee of College of Dentistry, University of Ha’il, Saudi Arabia, approved the protocol of this study (No.: H-2022-033. The participants provided their informed consent to partake in the study.

**Fig. 1:**
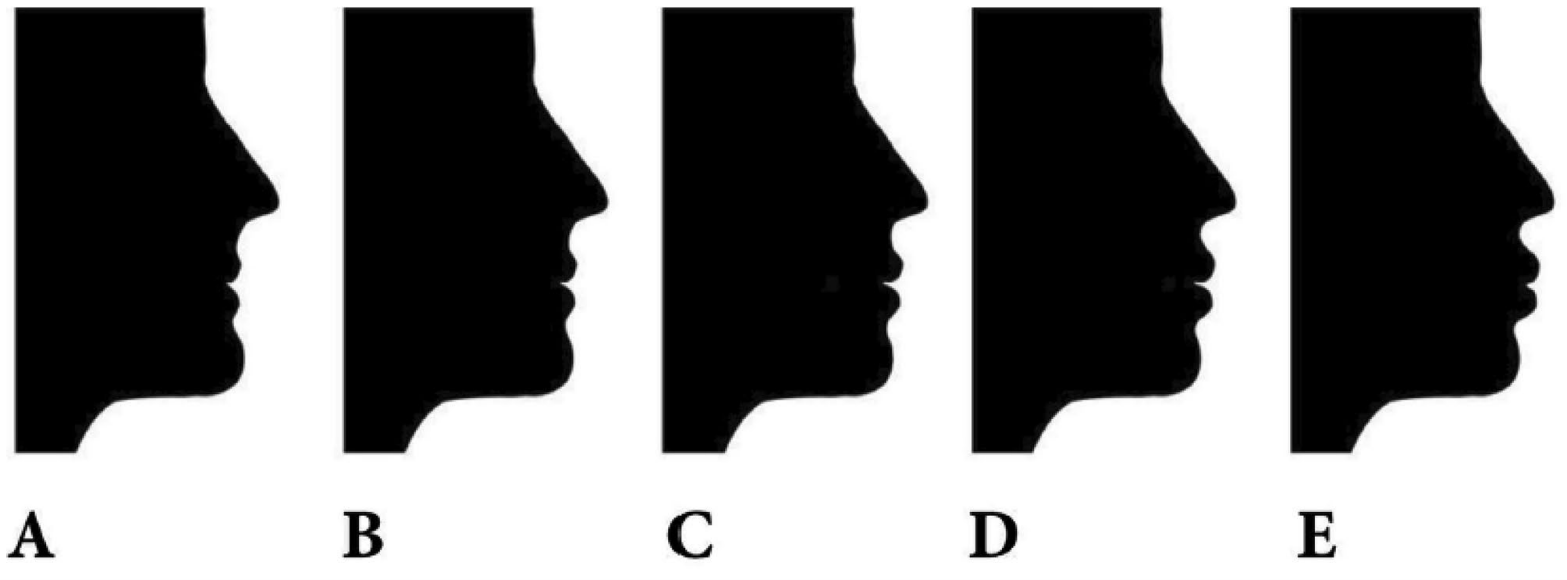
Displays the anterior divergent profiles, showcasing various lip positions. These positions are labeled as follows: **A** represents a lip retrusion of 4 mm, **B** represents a lip retrusion of 2 mm, **C** represents a normal lip position, **D** represents a lip protrusion of 2 mm, and **E** represents a lip protrusion of 4 mm. From Najafi et al. [30]

**Fig. 2:**
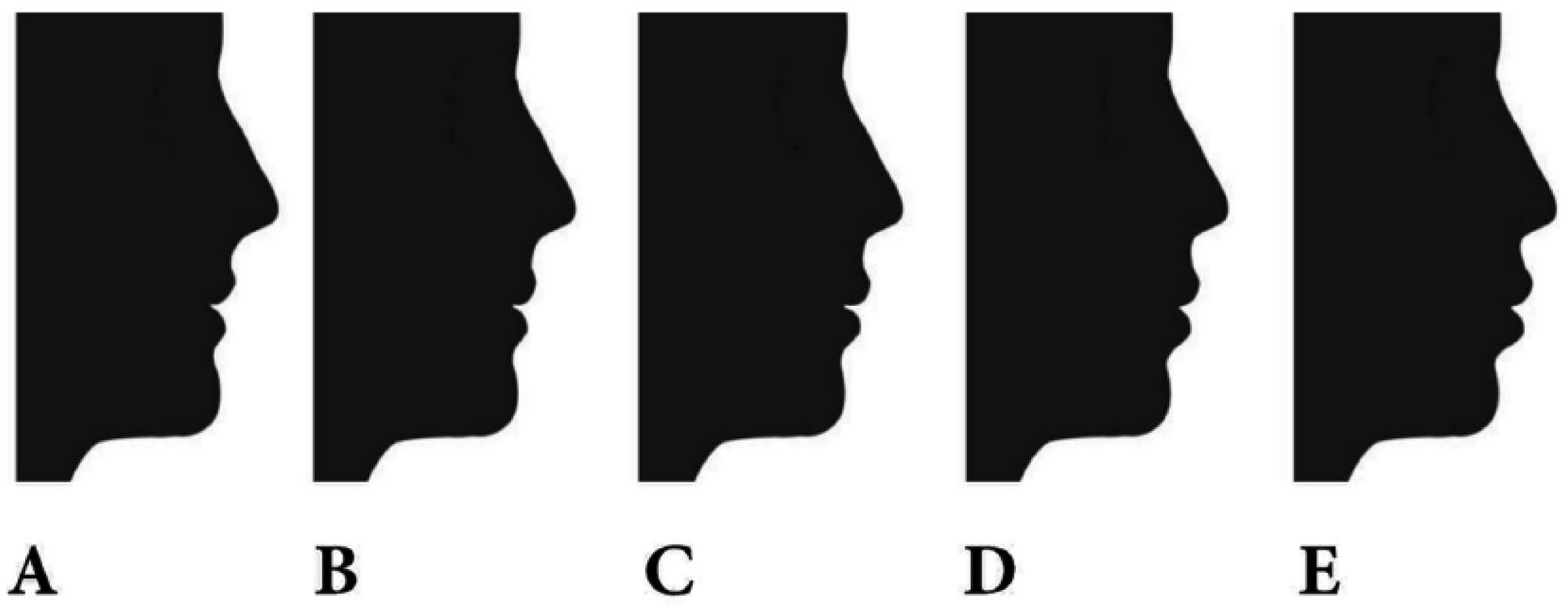
Displays the straight divergent profiles, showcasing various lip positions. These positions are labeled as follows: **A** represents a lip retrusion of 4 mm, **B** represents a lip retrusion of 2 mm, **C** represents a normal lip position, **D** represents a lip protrusion of 2 mm, and **E** represents a lip protrusion of 4 mm. From Najafi et al. [30]

**Fig. 3:**
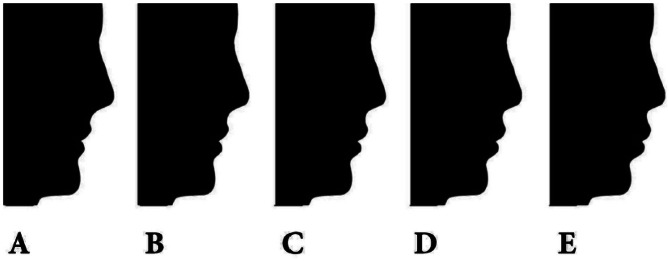
Displays the posterior divergent profiles, showcasing various lip positions. These positions are labeled as follows: **A** represents a lip retrusion of 4 mm, **B** represents a lip retrusion of 2 mm, **C** represents a normal lip position, **D** represents a lip protrusion of 2 mm, and **E** represents a lip protrusion of 4 mm. From Najafi et al. [30]

The research employed a profile image of black silhouettes in their natural head posture, which were acquired with authorization from the author [30]. The images were modified to exclude hair, thereby minimizing the impact of sex-related characteristics. The previous study [30] utilized Photoshop software to modify the ideal profile image by adjusting the horizontal positioning of the subnasale and soft tissue pogonion in relation to the true vertical line that passes through the glabella. This adjustment aimed to create three variations of a normal profile: anterior divergent (G-Sn to true vertical line + 15°, G-pog’ to true vertical line + 10°), straight divergent (G-Sn to true vertical line + 5°, G-pog’ to true vertical line 0°), and posterior divergent (G-Sn to true vertical line − 5°, G-pog’ to true vertical line − 10°). In order to focus exclusively on the sagittal face profile, no alterations were made to the vertical facial height profile. The profiles were categorized into three groups: straight, anterior divergent, and posterior divergent. Each group consisted of five photos depicting different levels of lip protrusion. In this study, each group consisted of five profiles that were assigned labels 1, 2, 3, 4, and 5, denoting various degrees of lip protrusion. The profile with the average lip protrusion was positioned in the middle of each series, as depicted in Figs. 1, 2 and 3. The labial region was further modified in 2 mm intervals to achieve varying degrees of protrusion or retraction, leading to an 8 mm disparity between the most prominent and the most recessed profiles within each set. The lip locations were consistently modified in the sagittal plane, yielding a set of 15 pictures exhibiting various combinations of face divergence and lip protrusion.

A group of laypeople from different parts of Saudi Arabia was selected using a non-probability snowball sampling technique to rate the profiles based on a 5-point Likert scale. In order to meet the eligibility criteria, participants were required to be at least 18 years of age, without any previous orthodontic or facial surgical interventions, devoid of any facial deformities or no facial trauma, and not employed in healthcare.

The data collection process involved the utilization of various social media communication channels, including Twitter and WhatsApp, to gather information. The data was obtained from diverse regions around Saudi Arabia. The study’s data gathering period spanned from January 1st, 2023, to March 30, 2023.

A Likert-type rating scale questionnaire was developed with the aim of assessing the profiles. The Likert scale is commonly referenced in literature as a valuable method for rating. The raters were obligated to evaluate each collection of profile series within a singular session and provide them a rating on a scale ranging from 1 to 5, where 1 represents a state of being highly unattractive and 5 signifies a state of being incredibly attractive. Furthermore, participants were explicitly advised against assigning identical numbers to many profiles. The questionnaire also contained demographic items pertaining to age, gender, monthly income, geographical region, and educational attainment. Every survey was assigned a distinct numerical code and ensured perfect anonymity. A pilot study was conducted to assess the precision, comprehensibility, and time constraints of the questionnaire.

The research included the analysis of categorical data, which was presented in a descriptive manner through the use of numerical values and percentages. The Pearson Chi Square test was employed to conduct inferential statistics, with a significance level of p < 0.05. The data underwent coding and recording within a Microsoft Excel file before being subsequently moved to the Social Sciences Statistical software for the purpose of analysis.

Statistical Package for the Social Sciences version 22 from IBM Co. was used for the data analysis, which included frequency distribution and cross-tabulation. The different lip locations and profile divergences were organized. The relationship between the demographic variables and questions related to aesthetics were analyzed using chi-square test. The threshold for significance was set at 5% (*p* < 0.05).

## Results

The study consisted of a total of 1077 individuals, of which 76.5% were identified as female, while the remaining participants were male. A total of 704 individuals of the sample (65.4%) were aged between 18 and 29 years. The patients aged 60 years and above exhibited the lowest level of representation (1.2%), while those between the ages of 30 and 39 were 20.3% of the study sample. In terms of geographical distribution, the central region garnered the highest responses (62.9%), while the northern and eastern regions accounted for 14.1% and 9%, respectively. The data reveals that a majority of the respondents, specifically 70.6%, possessed bachelor’s degrees, but a smaller proportion, namely 13.1%, held postgraduate degrees as shown in Table 1.

**Table 1:** Demographic Characteristics of the Participants

*Demographic Characteristics*	*Number of respondents (%)*	
Male	253	23.5%
Female	824	76.5%
Total	1077	100%
**Age in Years**		
18–29 Years	704	65.4%
30–39 years	219	20.3%
40–49 Years	117	10.9%
50–59 Years	24	2.2%
Above 60 Years	13	1.2%
Total	1077	100%
**Level of Education**		
Primary School	5	0.5%
Secondary School	12	1.1%
High School	159	14.8%
Bachelor’s degree	760	70.6%
Postgraduate Study	141	13.1%
Total	1077	100%
**Region**		
Central	677	62.9%
Western	82	7.6%
Eastern	97	9.0%
Sothern	69	6.4%
Northern	152	14.1%
Total	1077	100%

The analysis revealed differences among the three groups (Anterior divergent; straight divergent; posterior divergent) and within each group (A: 4-mm lip retrusion; B:2-mm lip retrusion; C: Normal lip position; D: 2-mm lip protrusion; E:4-mm lip protrusion) are shown in Table 2. In S1 (A and B), the most common aesthetic perception was neutral (40.2% and 36.4% respectively). In S1 (C), the highest frequency was for attractive aesthetics (31.4%), followed by neutral aesthetics (29.4%) and very attractive aesthetics (19.3%). In S1 (D), the highest frequency was for attractive aesthetics (27.4%). Conversely, in S1 (E), the highest frequency was for very unattractive aesthetics (37.3%). The mean ± S.D within each group ranged from 2.32 ± 1.33 [S1 (E)] to 3.44 ± 1.12 [S1 (C)]. Within the S2 (straight divergent) group, the highest frequency of aesthetics was observed in S2 (A), where very unattractive aesthetics had the highest frequency (31.7%). Similarly, in S2 (B) and S2 (C) unattractive aesthetics were the most common, with frequencies of 32.6% and 30.2% respectively. In S2 (D) and S2 (E), unattractive aesthetics also prevailed, with frequencies of 28.8% and 25.4% respectively. The S2 (E) had very attractive aesthetics in 10.9%. The mean ± S.D within each group ranged from 2.34 ± 1.22 [S2 (A)] to 2.60 ± 1.28 [S2 (E)]. Within the S3, the highest frequency of aesthetics was consistently very unattractive across all lip positions [S3 (A, B, C, D and E)], with frequencies of 66.5%, 54.4%, 50.9%, 49.5%, and 51.8%, respectively. The S3 (E) had very attractive aesthetics in only 7.1%. The mean ± S.D for lip positions ranges from 1.62 ± 1.06 [S3 (A)] to 1.97 ± 1.25 [S3 (E)].

**Table 2:** S1 Anterior divergent; S2 straight divergent; S3 posterior divergent; **A**: 4-mm lip retrusion; **B**: 2-mm lip retrusion; **C**: Normal lip position; **D**: 2-mm lip protrusion; **E**: 4-mm lip protrusion. 1 to 5, with 1 being very unattractive and 5 being very attractive

Profile image		1		2		3		4		5		Mean±S.D
**S1**	**A**	115	10.7%	240	22.3%	433	40.2%	176	16.3%	113	10.5%	2.94±1.10
	**B**	44	4.1%	193	17.9%	392	36.4%	263	27.2%	155	14.4%	3.30±1.05
	**C**	63	5.8%	151	14.0%	317	29.4%	338	31.4%	208	19.3%	3.44±1.12
	**D**	95	8.8%	230	21.4%	280	26.0%	295	27.4%	177	16.4%	3.21±1.20
	**E**	402	37.3%	257	23.9%	191	17.7%	121	11.2%	106	9.8%	2.32±1.33
**S2**	**A**	341	31.7%	298	27.7%	246	22.8%	113	10.5%	79	7.3%	2.34±1.22
	**B**	261	24.2%	351	32.6%	288	26.7%	123	11.4%	54	5.0%	2.40±1.12
	**C**	215	20.0%	325	30.2%	319	29.6%	142	13.2%	76	7.1%	2.57±1.15
	**D**	231	21.4%	310	28.8%	302	28.0%	155	14.4%	79	7.3%	2.57±1.18
	**E**	269	25.0%	274	25.4%	273	25.3%	144	13.4%	117	10.9%	2.60±1.28
**S3**	**A**	716	66.5%	193	17.9%	82	7.6%	37	3.4%	49	4.5%	1.62±1.06
	**B**	586	54.4%	301	27.9%	95	8.8%	47	4.4%	48	4.5%	1.77±1.07
	**C**	548	50.9%	268	24.9%	163	15.1%	51	4.7%	47	4.4%	1.87±1.10
	**D**	533	49.5%	275	25.5%	135	12.5%	72	6.7%	62	5.8%	1.94±1.18
	**E**	558	51.8%	231	21.4%	131	12.2%	80	7.4%	77	7.1%	1.97±1.25

The analysis of the correlation between demographic variables and the different profile esthetics are presented in Table 3. In the S1 group, there are significant differences were observed in the correlation between gender and S1 (A) or S1 (E) groups (*p* < 0.05). Regarding the relationship between age and the S1 profile, significant correlations were found in the S1 (B) and S1 (C) groups (*p* < 0.05). Additionally, significant differences were observed in the correlation between region and level of education in the S1 (B), S1 (C), and S1(D) groups (*p* < 0.05). However, no significant differences were found between monthly income and the (S1) across all lip positions (*p* > 0.05). In the S2 group, significant differences were identified between gender and the S2 (E) group (*p* < 0.05). Regarding the association between age and the S2 profile, significant correlations were found in the S2 (A), S2 (B), and S2 (C) groups (*p* < 0.05). Furthermore, highly significant differences were observed in the correlation between region and level of education in the S2 (D) group (*p* < 0.05). However, no significant differences were detected between monthly income and the S2 group across all lip positions (*p* > 0.05). In the S3 group, highly significant differences were only found between gender and the S3E group (*p* < 0.005). Regarding the association between age and the S3 profile, highly significant correlations were found in the S3 (D) and S3 (E) groups (*p* < 0.005). Furthermore, significant differences were observed in the correlation between region and level of education in the S3 (C) and S3 (D) groups (*p* < 0.05). In contrast to the S1 and S2 groups, monthly income showed significant differences in the S3 (A), S3 (B), and S3 (E) groups (*p* < 0.05).

**Table 3:** Relationship between demographic variables and questions related to aesthetics

Questions Variables		Gender	Age	Region	Level of Education	Monthly Income
**S1**	**A**	0.004	0.90	0.17	0.17	0.50
	**B**	0.28	0.01	0.01	0.01	0.66
	**C**	0.59	0.03	0.000	0.00	0.58
	**D**	0.92	0.06	0.04	0.04	0.51
	**E**	0.008	0.10	0.06	0.06	0.78
**S2**	**A**	0.29	0.05	0.13	0.13	0.41
	**B**	0.13	0.000	0.32	0.32	0.30
	**C**	0.25	0.008	0.22	0.22	0.36
	**D**	0.17	0.28	0.005	0.005	0.75
	**E**	0.003	0.95	0.98	0.98	0.62
**S3**	**A**	0.23	0.30	0.60	0.60	0.04
	**B**	0.13	0.08	0.08	0.08	0.02
	**C**	0.17	0.08	0.01	0.01	0.07
	**D**	0.06	0.002	0.05	0.05	0.52
	**E**	0.000	0.000	0.007	0.007	0.01

## Discussion

The findings of this study indicate that aesthetic perceptions exhibited variability across various lip positions and profile divergences. Within the anterior diverging group, it was seen that the lip position most commonly favored was within the neutral range. Additionally, it was shown that aesthetics deemed attractive were predominantly associated with the normal lip position. Nevertheless, when the degree of lip protrusion either grew or reduced, there was a noticeable shift in aesthetic evaluations towards ugly or highly unpleasant aesthetics. Within the category of straight diverging individuals, it was shown that unappealing visual qualities were more commonly detected in all lip positions. However, notably pleasant visual qualities were observed specifically in the lip position that protruded the greatest, measuring 4 millimeters. Within the posterior divergent group, a consistent observation was made about the presence of unpleasant aesthetics across all lip locations, with the exception of the most protruded lip position (4-mm lip protrusion), which exhibited highly attractive aesthetics.

Recent research have revealed noteworthy findings on the esthetic evaluation of lip position in different profile divergences. The lip position in the anterior diverging profile, which is commonly determined using Ricketts’ values, was found to be in agreement with the findings of Najafi et al.‘s study [12, 30]. Nevertheless, the results of our own research exhibited different findings for the groups categorized as straight and posterior diverging. In these instances, it was seen that a lip protrusion of 4 mm, which deviates from Ricketts’ criteria and the findings of Najafi et al. [30] was linked to highly appealing aesthetics. Additionally, additional research [31, 32] have corroborated our findings, suggesting that individuals of both genders have a preference for a more pronounced lip position in contrast to the established criteria set by Ricketts [12]. Interestingly, orthodontic professionals have found that the typical lip position, as defined by Ricketts’ standards, is deemed the most aesthetically pleasing. In contrast, young undergraduate students tend to prefer slightly protrusive lip positions [32]. Milutinovic et al. [4] have observed that orthodontists, dentists, and plastic surgeons commonly hold the belief that a more prominent lip and a more symmetrical facial profile are regarded as the most visually appealing facial attributes. However, a study conducted on the Korean population revealed that those with protruding profiles were assigned lower ratings, whilst those with retruded profiles were favoured [19]. The observed disparity can be ascribed to disparities in professional education and exposure to Western influences, whereby orthodontic experts or trainees are more familiar with the distinctive facial features commonly associated with individuals of Caucasian descent [32]. An additional noteworthy finding was that individuals who did not receive orthodontic treatment exhibited a tendency to favor bigger lip positions in comparison to those who underwent such treatment [31]. In order to mitigate potential bias, our study deliberately selected individuals who lacked prior exposure to orthodontic treatment, primarily targeting laypersons. With the ongoing process of modernity and the gradual erosion of cultural boundaries, there is an increasing inclination among the general populace towards broader and more forwardly positioned lips, accompanied by a more pronounced nasolabial angle [33, 34]. In light of the dynamic nature of aesthetic preferences, it is recommended that although principles such as Ricketts’ E-line may continue to offer valuable insights for orthodontic treatment planning, it is imperative to also include modern aesthetic preferences [32]. The increasing prevalence of broader and more anteriorly positioned lips warrants careful consideration, particularly given the growing prominence of these trends within the general population.

The examination of the relationship between demographic characteristics and aesthetic perceptions yielded intriguing results. The influence of gender on aesthetic perceptions was shown to be significant in the anterior divergent group (with 4-mm lip retrusion and 4-mm lip protrusion), the straight divergent group (with 4-mm lip protrusion), and the posterior divergent group (with 4-mm lip protrusion). This discovery is consistent with a prior investigation that similarly observed gender disparities in aesthetic assessments of the anterior diverging profile featuring a 4-mm lip retrusion and the anterior divergent profile including a 2-mm lip protrusion [30]. The result is consistent with earlier research that has shown a significant disparity in the perception of smiling between males and females [2]. The current study specifically indicated that the female profile exhibited lower average scores compared to the male profile in relation to the 4-mm retruded lip position.

This finding coincides with other research that has indicated a general preference for larger lips among females as compared to males [35–37]. Nevertheless, Shimomura and colleagues [38] made the notable finding that orthodontic patients had a tendency to favor a slightly retruded lip position as compared to an average facial profile, with a greater inclination towards this preference observed in female profiles. In this study, the exclusion criteria encompass orthodontic patients and healthcare providers, as a means to regulate the influence of profile preferences. The diverse outcomes seen in this study can be ascribed to the impact of cultural influence, background, and ethnic norms on individuals’ profile preferences [20, 39–41].

The impression of lip profiles that are acceptable for one’s age is a significant determinant of both face attractiveness and social acceptance. The findings of our investigation indicate that the younger participants had a predilection for top and lower lips that protrude, which is consistent with the existing literature suggesting a prevalent preference among young people for lips that are fuller [32]. In our study, it was seen that individuals belonging to the middle and older age groups exhibited a preference for a lip position that was either normal or somewhat retruded, as indicated by the E-line values proposed by Ricketts. The variation in preference can be ascribed to age-related alterations in facial characteristics and the impression of a lip profile that is considered aesthetically pleasant. The preferences for lip profiles in individuals can be influenced by various factors such as cultural standards, societal trends, and changes in face characteristics that occur with aging [34]. Chan et al. [42] observed a clear inclination towards flatter lip profiles among Asian Chinese individuals, potentially affected by cultural norms and unique aesthetic standards prevalent in their society. A further significant determinant impacting the choice for lip profile is the comparative alteration in lip positioning in relation to other facial attributes as individuals undergo the aging process. According to Bishara et al. (year), their research revealed that as individuals age, there is a tendency for the lips to have a more retruded appearance in comparison to the chin and nose. As a result, it is possible that individuals in the middle and older age groups may exhibit a tendency towards a retruded lip profile [43]. Shimomura and colleagues (year) made an additional observation that female patients of Japanese descent who were older than 30 years exhibited a greater preference for a lip position that is more retruded when compared to younger age cohorts. The aforementioned findings underscore the notable influence of cultural and age-related variables on the formation of judgments about lip profiles [38].

Furthermore, there were notable associations observed between aesthetic evaluations and both geographic location and educational attainment in relation to specific lip positions within the anterior divergent (2-mm lip retrusion, Normal lip position, and 2-mm lip protrusion), straight divergent (2-mm lip protrusion), and posterior divergent (Normal lip position and 2-mm lip protrusion) categories. In contrast, the monthly income variable did not exert a statistically significant impact on aesthetic assessments within any of the lip positions across all demographic groups. The aforementioned findings underscore the significance of taking into account individual preferences and demographic variables when assessing the aesthetics of lip position and profile.

The research findings illustrate that aesthetic perceptions may exhibit variability contingent upon factors such as gender, age, geographical location, and educational attainment. Previous research has indicated that there exists a significant degree of variation in the perception of aesthetics when it comes to smiles. Furthermore, factors such as gender, age, and prior experience with dental treatments exhibit a considerable influence on individuals’ impression of smile aesthetics [3]. These elements might play a role in shaping cultural and societal norms surrounding beauty standards and aesthetic preferences. A comprehensive comprehension of these variances can assist orthodontists and maxillofacial surgeons in customizing treatment programs to align with the expectations and desires of their patients.

It is worth noting that this study had some limitations. The use of black silhouettes in the evaluation of lip position and profile divergence may not fully capture the complexity and nuances of facial aesthetics. However, for evaluating profile aesthetics, previous authors have suggested using silhouettes designs since they minimize the impact of other aesthetic factors like hair, skin, and eyes [37, 44]. Additionally, the study sample consisted of laypeople from Saudi Arabia, which may limit the generalizability of the findings to other populations. Further research with larger and more diverse samples is needed to validate and expand upon these findings.

## Conclusions

Under the limitations of this study, it can be concluded that aesthetic preferences varied based on the positioning of the lips and the level of divergence in the facial profile. Distinct variations were seen among the groups classified as anterior, straight, and posterior divergent. Significant factors that were shown to have an influence on aesthetic impressions include gender, age, area, and degree of education. Clinicians are advised to customize the treatment plan in accordance with the patient’s particular desires and preferences.

## Data Availability

The datasets created and/or analyzed for the current study are not publicly accessible because ethics approval was given on the grounds that only the researchers involved in the study would have access to the identified data, but they are available from the corresponding author upon justifiable request.

## References

[1] Bonetti GA, Alberti A, Sartini C, Parenti SI (2011). Patients’ self-perception of dentofacial attractiveness before and after exposure to facial photographs. Angle Orthod.

[2] Pasukdee P, Cheng JHC, Chen DS (2021). Smile preferences of orthodontics, general dentists, patients, and the general public in three-quarter and lateral views. Am J Orthod Dentofacial Orthop.

[3] Cheng JH, Hsu YC, Lee TY, Li RW (2023). Factors affecting perception of laypeople and dental professionals toward different smile esthetics. J Dent Sci.

[4] Milutinovic J, Aleksic E, Avramov S, Kalevski K, Gajic M, Pejanovic D, Milic J (2023). Esthetic preferences of orthodontists, dentists, and plastic surgeons for balanced facial profiles. J Oral Sci.

[5] Tufekci E, Jahangiri A, Lindauer SJ (2008). Perception of profile among laypeople, dental students and orthodontic patients. Angle Orthod.

[6] Hicks KE, Thomas JR (2020). The changing face of beauty: a global assessment of facial beauty. Otolaryngol Clin N Am.

[7] Mousavi SM, Saeidi Ghorani P, Deilamani A, Rakhshan V (2019). Effects of laterality on esthetic preferences of orthodontists, maxillofacial surgeons, and laypeople regarding the lip position and facial convexity: a psychometric clinical trial. Oral Maxillofac Surg.

[8] Sukhia RH, Khan M, Fida M, Shaikh A, Azam SI (2011). Esthetic preferences for facial soft tissue profiles. Int J Orthod Milwaukee.

[9] Alrbata RH, Alfaqih AK, Almhaidat MR, Al-Tarawneh AM. Thresholds of abnormality perception in facial esthetics among laypersons and dental professionals: profile esthetics. Int J Dent. 2020. 2068961. 10.1155/2020/2068961 PMC7568134 33101413

[10] Thakral R, Kapoor S, Shukla P, Sharma VK, Bhagchandani J, Agarwal S (2022). Perception of facial esthetics in young north Indian population. J Orthodont Sci.

[11] Steiner CC (1959). Cephalometrics in clinical practice. Angle Orthod.

[12] Ricketts RM (1968). Esthetics, environment, and the law of lip relation. Am J Orthod.

[13] Cheng JHC, Luechapanichkul MJ, Lee TYH (2021). The relationship between dentofacial morphology and smile characteristics in lateral and oblique views. J Dent Sci.

[14] Naini FB, Cobourne MT, McDonald F, Garagiola U, Wertheim D (2021). Quantitative investigation of the esthetic impact of lip prominence in relation to the esthetic line. Am J Orthod Dentofacial Orthop.

[15] Matoula S, Pancherz H (2006). Skeletofacial morphology of attractive and nonattractive faces. Angle Orthod.

[16] Nomura M, Motegi E, Hatch JP, Gakunga PT, Ng’ang’a PM, Rugh JD, Yamaguchi H (2009). Esthetic preferences of European American, Hispanic American, Japanese, and African judges for soft-tissue profiles. Am J Orthod Dentofacial Orthop.

[17] Khosravanifard B, Rakhshan V, Raeesi E (2013). Factors influencing attractiveness of soft tissue profile. Oral Surg Oral Med Oral Pathol Oral Radiol Endod.

[18] Ding A (2021). The ideal lips: lessons learnt from the literature. Aesthet Plast Surg.

[19] Seo KH, So DH, Song KT, Choi SK, Kang KH (2021). Effect of lower facial height and anteroposterior lip position on esthetic preference for Korean silhouette profiles. Korean J Orthod.

[20] Maple JR, Vig KW, Beck FM, Larsen PE, Shanker S (2005). A comparison of providers’ and consumers’ perceptions of facial-profile attractiveness. Am J Orthod Dentofacial Orthop.

[21] Bravo LA, Canut JA, Pascual A, Bravo B (1997). Comparison of the changes in facial profile after orthodontic treatment, with and without extractions. Br J Orthod.

[22] Zhang Y-f, Xiao L, Li J, Peng Y-r, Zhao Z (2010). Young people’s esthetic perception of dental midline deviation. Angle Orthod.

[23] Mohammed A, Shetty A, Nayak UK, Shetty P, Mendonca M, Shashidhar K (2017). Perception of facial profile attractiveness by orthodontists and general public in Dakshina Kannada population. J Health Allied Sci NU.

[24] Aldelaimi TN, Khalil AA (2014). Lip reconstruction using Karapandzic flap. J Craniofac Surg.

[25] Aldelaimi TN, Khalil AA (2015). Reconstruction of facial defect using deltopectoral flap. J Craniofac Surg.

[26] Aldelaimi AA, Ahmed RF, Enezei HH, Aldelaimi TN (2019). Gummy smile esthetic correction with 940 nm Diode Laser. Int Med J.

[27] Gomes P, Jardim L (2006). Estudo cefalométrico do perfil cutâneo de jovens adultos tratados ortodonticamente com e sem extrações. Rev Port Estom Med Dent Cir Maxilofac.

[28] Lai J, Ghosh J, Nanda RS (2000). Effects of orthodontic therapy on the facial profile in long and short vertical facial patterns. Am J Orthod Dentofacial Orthop.

[29] Boneco C, Jardim L (2005). Estudo Da Morfologia labial em pacientes com padrão facial vertical alterado. Rev Port Estom Med Dent Cir Maxilofac.

[30] Najafi HZ, Sabouri SAA, Ebrahimi E, Torkan S (2016). Esthetic evaluation of lip position in silhouette with respect to profile divergence. Am J Orthod Dentofacial Orthop.

[31] Hier LA, Evans CA, BeGole EA, Giddon DB (1999). Comparison of preferences in lip position using computer animated imaging. Angle Orthod.

[32] Otuyemi O, Afolabi D, Oyewole T (2022). Ricketts’ E-line profile preferences among Nigerian orthodontists, orthodontic trainees, and a young undergraduate students’ population. Niger J Clin Pract.

[33] Auger T, Turley PK (1999). The female soft tissue profile as presented in fashion magazines during the 1900s: a photographic analysis. Int J Adult Orthodon Orthognath Surg.

[34] Yehezkel S, Turley PK (2004). Changes in the African American female profile as depicted in fashion magazines during the 20th century. Am J Orthod Dentofacial Orthop.

[35] Foster EJ (1973). Profile preferences among diversified groups. Angle Orthod.

[36] Czarnecki ST, Nanda RS, Currier GF (1993). Perceptions of a balanced facial profile. Am J Orthod Dentofacial Orthop.

[37] Coleman GG, Lindauer SJ, Tüfekçi E, Shroff B, Best AM (2007). Influence of chin prominence on esthetic lip profile preferences. Am J Orthod Dentofacial Orthop.

[38] Shimomura T, Ioi H, Nakata S, Counts AL (2011). Evaluation of well-balanced lip position by Japanese orthodontic patients. Am J Orthod Dentofacial Orthop.

[39] Mejia-Maidl M, Evans CA, Viana G, Anderson NK, Giddon DB (2005). Preferences for facial profiles between Mexican americans and caucasians. Angle Orthod.

[40] Samizadeh S, Wu W (2020). Ideals of facial beauty amongst the Chinese population: results from a large national survey. Aesthet Surg J.

[41] Todd S-A, Hammond P, Hutton T, Cochrane S, Cunningham S (2005). Perceptions of facial aesthetics in two and three dimensions. Eur J Orthod.

[42] Chan EK, Soh J, Petocz P, Darendeliler MA (2008). Esthetic evaluation of asian-Chinese profiles from a white perspective. Am J Orthod Dentofacial Orthop.

[43] Bishara SE, Treder JE, Jakobsen JR (1994). Facial and dental changes in adulthood. Am J Orthod Dentofacial Orthop.

[44] Spyropoulos MN, Halazonetis DJ (2001). Significance of the soft tissue profile on facial esthetics. Am J Orthod Dentofacial Orthop.

